# LPPR5 Expression in Glioma Affects Growth, Vascular Architecture, and Sunitinib Resistance

**DOI:** 10.3390/ijms23063108

**Published:** 2022-03-13

**Authors:** Lena Stange, Kristin Elizabeth Lucia, Adnan Ghori, Peter Vajkoczy, Marcus Czabanka, Thomas Broggini

**Affiliations:** 1 Department of Neurosurgery, University Hospital Frankfurt, 60528 Frankfurt am Main, Germany; lena.stange@kgu.de (L.S.); kristin.lucia@kgu.de (K.E.L.); marcus.czabanka@kgu.de (M.C.); 2 Department of Neurosurgery, Charité-Universitätsmedizin Berlin, 10117 Berlin, Germany; adnan.ghori@charite.de (A.G.); peter.vajkoczy@charite.de (P.V.)

**Keywords:** plppr5, lppr5, PRG5, antiangiogenic therapy resistance, sunitinib

## Abstract

Despite intensive research, glioblastoma remains almost invariably fatal. Various promising drugs targeting specific aspects of glioma biology, in addition to or as an alternative to antiproliferative chemotherapy, were not successful in larger clinical trials. Further insights into the biology of glioma and the mechanisms behind the evasive-adaptive response to targeted therapies is needed to help identify new therapeutic targets, prognostics, or predictive biomarkers. As a modulator of the canonically oncogenic Rho-GTPase pathway, Lipid phosphate phosphatase-related protein type 5 (LPPR5) is pivotal in influencing growth, angiogenesis, and therapeutic resistance. We used a GL261 murine orthotopic allograft glioma model to quantify the tumor growth and to obtain tissue for histological and molecular analysis. Epicortical intravital epi-illumination fluorescence video microscopy of the tumor cell spheroids was used to characterize the neovascular architecture and hemodynamics. GL261-glioma growth was delayed and decelerated after LPPR5 overexpression (LPPR5^OE^). We observed increased tumor cell apoptosis and decreased expression and secretion of vascular endothelial growth factor A in LPPR5^OE^ glioma. Hence, an altered micro-angioarchitecture consisting of dysfunctional small blood vessels was discovered in the LPPR5^OE^ tumors. Sunitinib therapy eliminated these vessels but had no effect on tumor growth or apoptosis. In general, LPPR5 overexpression generated a more benign, proapoptotic glioma phenotype with delayed growth and a dysfunctional vascular architecture.

## 1. Introduction

Glioblastoma, the most frequent and most malignant primary brain tumor, develops either de novo or from a lower grade glioma (LGG) precursor lesion [1,2,3]. The main pathophysiological characteristics of glioblastoma include necrotic cores, high tumor cell invasion, and chaotic, dysfunctional vasculature. The stratification of histologically comparable tumors is based on molecular features, in particular IDH mutation and the MGMT-promotor methylation status [3,4,5]. Targeted therapies, as an alternative or in addition to traditional cytotoxic and anti-proliferative chemotherapy, have been deployed, with disappointing outcomes in most patients [6,7,8]. Further insights into the biology of glioma and the mechanisms behind the evasive response to these therapies may help identify new therapeutic targets, prognostics, or predictive biomarkers.

A promising candidate is LPPR5, an integral membrane protein linked to neuronal plasticity which impedes NogoA- and LPA-mediated RhoA kinase signaling [9]. LPPR5 modulates the Rho–GTPase pathway involved in cancer growth, vascularization, and the adaptive response to changes in the microenvironment [10]. The role of LPPR5 in neoplasia is currently unknown, although LPPR5 is almost exclusively expressed in the tissue of the central nervous system, and the gene is located on the 1p allele. This locus is commonly deleted in oligodendroglioma with unknown biological significance [11]. Rho GTPases are considered pro-oncogenic facilitators of the Ras family, and LPPR5 activity has been shown to impede RhoA signaling [9]. We therefore hypothesized that high LPPR5 expression will exert anti-neoplastic effects in glioma.

## 2. Results

### 2.1. In Silico Analysis in Human Diffuse Glioma

**Figure 1:**
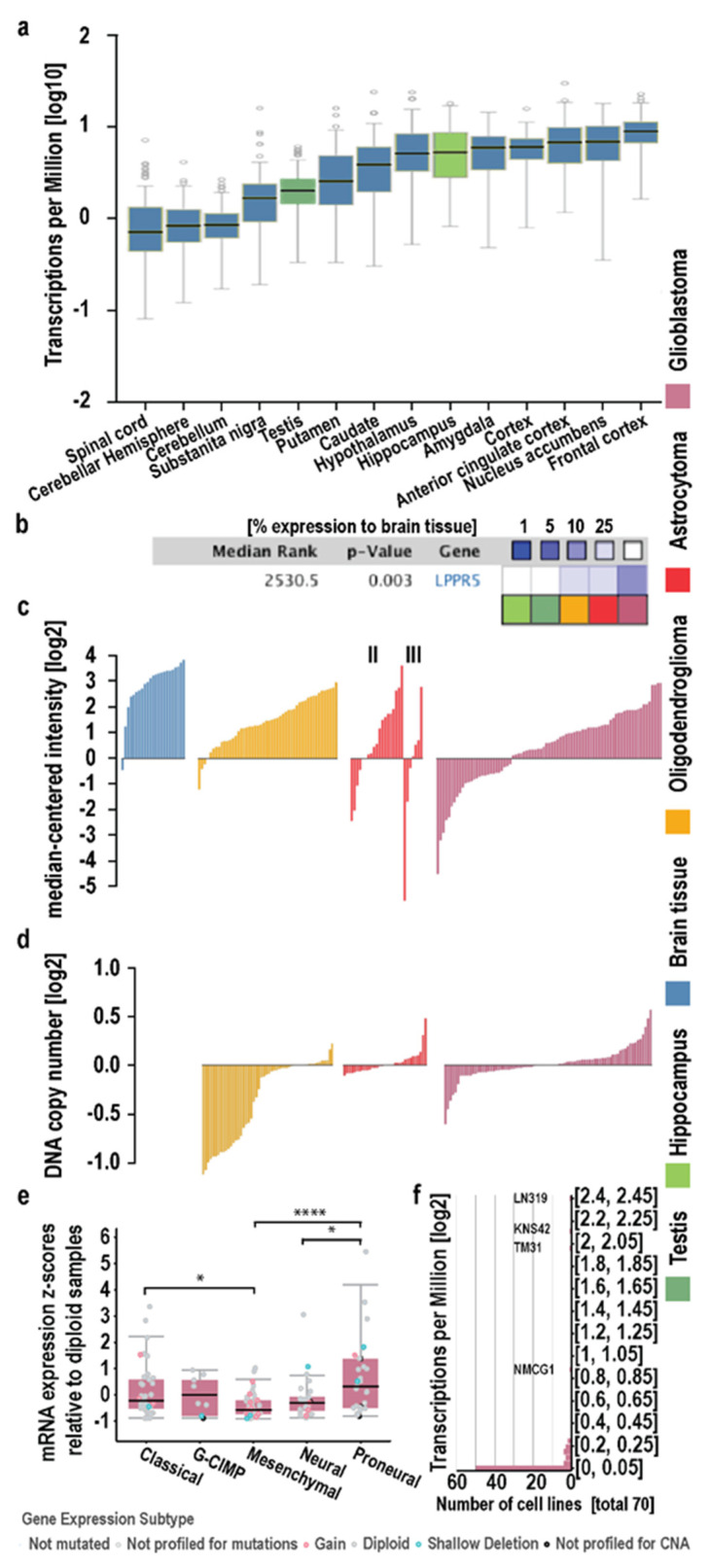
In-silico analysis of LPPR5 in physiological brain tissue and primary brain tumor. (**a**) LPPR5 expression is physiologically high in human adult brain. Outside the CNS, LPPR5 is compara-tively highly expressed in testis [GTeX gene expression data for LPPR5 in brain (blue, Hippocampus light green) and non-brain tissue (dark green). Expression values are shown in TPM (Tran-scripts Per Million), calculated from a gene model with isoforms collapsed to a single gene. No other normalization steps have been applied. Box plots are shown as median and 25th and 75th per-centiles; points are displayed as outliers if they are above or below 1.5 times the interquartile range]. (**b**) LPPR5 is significantly un-der-expressed in (high-grade) glioma. Relative under-expression rank is median rank across all analyses in gene expression in Sun Brain, and respectively Roth normal in Oncomine [p for medi-an-ranked analysis, Oncomine visualization and statistical anal-ysis]. (**c**) A subgroup of high-grade glioma has decreased LPPR5 expression; in lower grade glioma, the ratio of tumors with low LPPR5 expression is smaller [mRNA expression data from Sun Brain, each bar represents a single tumor, II and III indicates astrocytoma WHO grade]. (**d**) On the DNA-level, LPPR5 is deleted in most oligodendrogliomas and to a lesser degree in lower grade astrocytic gliomas and GBM [Oncomine visualization of LPPR5 DNA copy number units of the tumors in Kotliarov Brain, each bar represents a single tumor]. (**e**) LPPR5 expression is significantly lower in the mesenchymal expression subtype of GBM (*n* = 49) compared to classical (*n* = 39, * *p* = 0.0451) and proneural (*n* = 29, **** *p* < 0.0001) subtypes. Expression in the neural subtype (*n* = 26) is significantly lower than in proneural subtype (* *p* = 0.0162) [TCGA via cBio]. (**f**) Histogram plot of 70 human Glioma cell lines screened, 50 show low expression of LPPR5 [Cancer Cell Line Encyclopedia, Broad Institute].

The strongest expression of LPPR5 was found in different regions of the central nervous system (CNS) and testis in physiological conditions (Figure 1a and Figure S1). We compared the physiological expression in the testis (dark green) and hippocampus (light green) to the expression levels found in the oligodendroglioma (yellow), astrocytoma (red), and glioblastoma (purple) in the Sun cohort (Figure 1b) [12]. We found the expression of LPPR5 was reduced in neoplasia with higher WHO classifications (Figure 1b,c). We explored this phenomenon on an individual level in different diffuse glioma entities and found a higher fraction of glioblastoma tumors with downregulated LPPR5 expression compared with other neoplasia (Figure 1c). We evaluated the DNA copy number inserts and deletions in these pathological tumors to determine the genetic origin of this downregulation. Deletion of LPPR5 is common in but not exclusive to oligodendrogliomas. However, the reduced expression after haploid loss of LPPR5 is readily overcompensated by the functional allele in oligodendrogliomas (Figure 1c,d). In the TCGA GBM cohort, copy number inserts and deletions were reported in a minority of the glioma. It is worth noting that the TCGA data available on GBM consists primarily, but not exclusively, of non-IDH-mutated glioblastomas and does not include methylation classifier data in accordance with the 2016 or 2021 WHO classification. The Sun and Kotliarov datasets provide data on the gene copy number variations and gene expression of a highly overlapping set of glioma samples, while the IDH status is unclear. Again, the most malignant mesenchymal glioma subtype showed the lowest expression levels of LPPR5 in the TCGA GBM cohort (Figure 1e). Moreover, in a screening of 70 human glioma cell lines from the Cancer Cell Line Encyclopedia (Broad Institute), only 4 cell lines showed a significant expression of LPPR5 (LN319, KNS42, TM31, and NMCG1; Figure 1f).

### 2.2. Characterization of LPPR5 Expression in GL261

**Figure 2:**
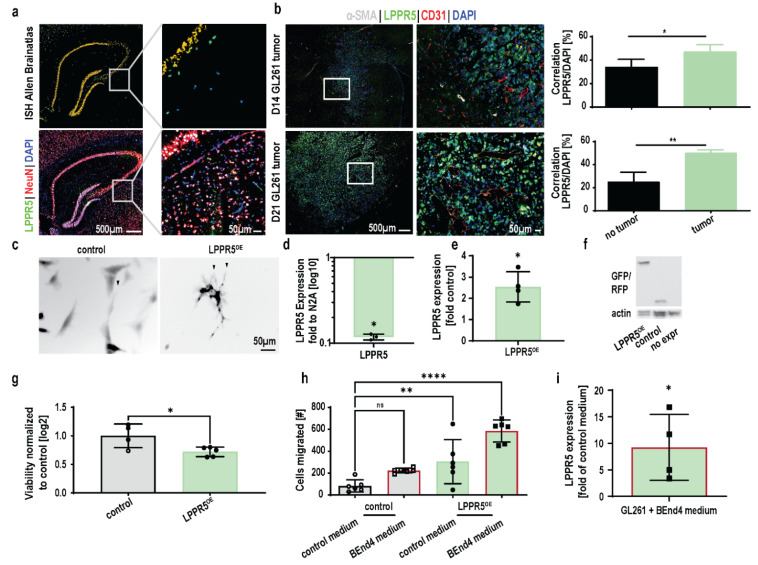
Characterization of LPPR5 expression in tumor cells. (**a**) Comparison between in-situ hybridization and immunohistochemical staining of LPPR5 in the murine hippocampus. Colocalization with the neuronal marker NeuN was observed (white signal, orange signal Figure S2). (**b**) Immunohistochemical staining of LPPR5 in GL216 murine glioma, 14 and 21 days after intracranial injection [aSMA indicates mature, functional blood vessel, CD31 labels blood vessels, DAPI labels nuclei]. Spatial image correlation of DAPI and LPPR5 signals shows increased cross correlation in tumor tissue compared to non-tumor tissue on the contralateral hemisphere 14 and 21 days after tumor cell injection [Student’s unpaired *t* test, 14 days * *p* = 0.0242, 21 days ** *p* = 0.0012]. (**c**) Overexpression of LPPR5 in-vitro shows an increase in filopodia formation in GL261 glioma cells (arrow heads). (**d**) qPCR of LPPR5 expression in GL261 was reduced to 0.11 compared to differentiated N2A cells [Student’s unpaired *t* test * *p* = 0.0173]. (**e**) qPCR of LPPR5 overexpressing clones show a 2.5-fold increase compared to control GFP clones [Student’s unpaired *t* test * *p* = 0.0416]. (**f**) Immunoblot of LPPR5 overexpression, control RFP and native GL261 shows the increase in size of the LPPR5-RFP fusion protein. (**g**) CELLigence RTCA viability assay display a reduced viability of LPPR5^OE^ GL261 during a 24-h observation period [Student’s unpaired *t* test * *p* = 0.027]. (**h**) CELLigence RTCA migration assay reveals increased trans well migration of LPPR5^OE^ clones (green bar, ** *p* = 0.0097) and an additional increase in migration towards endothelial cell conditioned medium (green bar with red border, **** *p* < 0.0001) [one-way ANOVA with Dunnett’s multiple comparisons test]. (**i**) Strong increase in LPPR5 expression 24h after direct application of endothelial cell conditioned medium on GL261 glioma cells [Student’s unpaired *t* test * *p* = 0.0081]. ns = non significant.

The Allen Brain Atlas identified in situ hybridization of LPPR5 mRNA in the hippocampal formation (Figure 2a). Immunohistochemical staining of LPPR5 (green) and NeuN (red) showed a spatially similar expression pattern in the hippocampal formation (Figure 2a and showed in more detail in Figure S2).

Strong increase in LPPR5 expression 24 h after direct application of endothelial cell conditioned medium on GL261 glioma cells [Student’s unpaired *t* test *p* = 0.0081]. An increase in correlation between the DAPI cell nuclei staining and the LPPR5 signal was observed in the GL261 murine brain tumors. This was significant 14 days post tumor cell injection and increased further with tumor progression (Figure 2b). LPPR5 was identified in neurons as a transmembrane protein, and the increased nuclear localization indicated an impairment of LPPR5 trafficking and possible accumulation of LPPR5 in the endoplasmic reticulum (Figure 2b).

In vitro overexpression of LPPR5 in GL261 induced morphological changes similar to the changes described in other cells previously (arrowheads in Figure 2c) [9]. Expression verification with qPCR found a reduction in LPPR5 expression in GL261 compared with the N2A murine neuroblastoma cells and a twofold increase in LPPR5 transcription in the overexpression clones (Figure 2d,e). Even though we fused LPPR5 with a fluorescent protein for spatial visualization, we performed immunoblotting to control for the protein size (Figure 2f). A CELLigence RTCA viability assay showed a reduction in cell viability in the LPPR5^OE^ clones (Figure 2g). The cell motility of the LPPR5^OE^ clones was increased in the CELLigence RTCA trans-well chamber migration assay. The endothelial cell conditioned medium (BEnd.4 medium) further increased the tumor cell motility of the LPPR5^OE^ clones (Figure 2h). Furthermore, the endothelial cell conditioned medium applied in vitro to control the GL261 cells led to a strong modulation of the expression levels of LPPR5 within 24 h (Figure 2i).

### 2.3. Growth of LPPR5^OE^ Glioma Is Decelerated and Reduced

**Figure 3:**
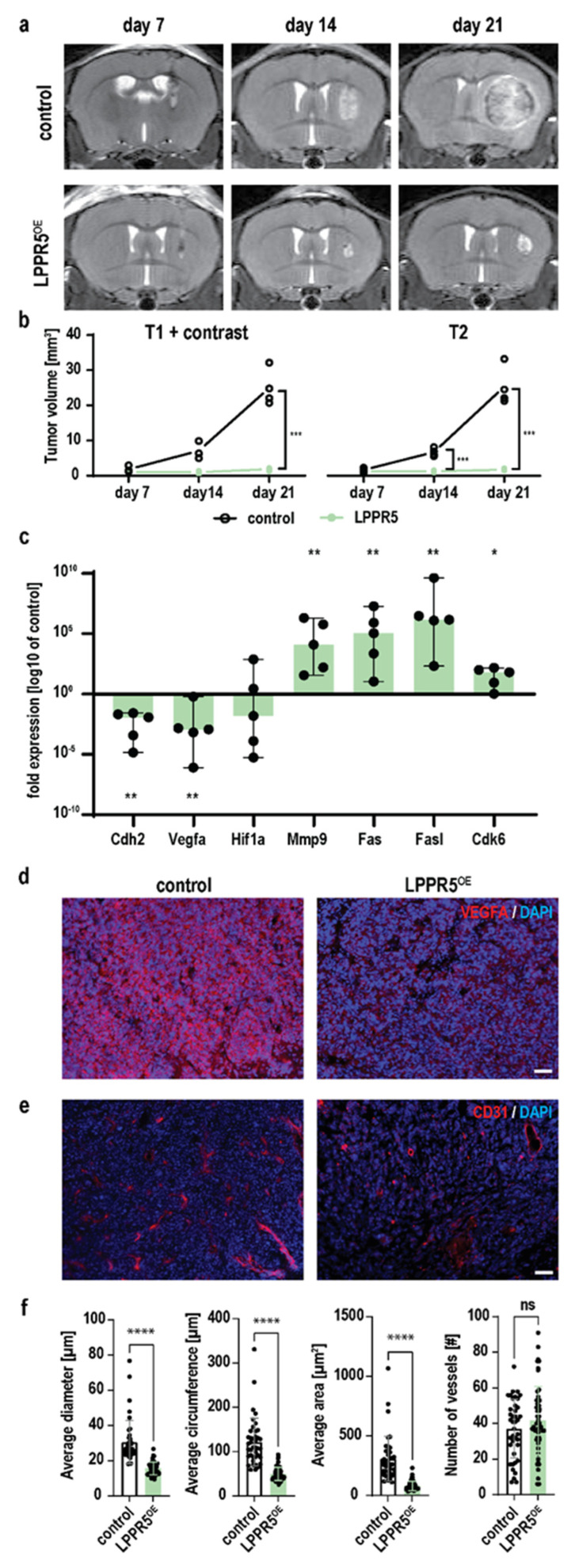
In-vivo characterization of LPPR5^OE^ tumors. (**a**) Coronal T2 MR images of tumor baring mice 7-, 14- and 21-days post tumor cell injection of control GL261 and LPPR5^OE^ clones. (**b**) T1 contrast and T2 MR image quantification shows higher sensitivity for volumetry with T2 sequences in these tumors [T1+contrast; day 21 *** *p* = 0.0080, T2; day 14 *** *p* = 0.0045, day 21 *** *p* = 0.0097, mixed effect analysis, Sidak’s multiple comparisons test]. (**c**) qPCR expression screening of apoptotic and angiogenic genes in LPPR5^OE^ tumors compared to control tumors [median and range of the values is plotted as whiskers. ** *p* = 0.008, * *p* = 0.032, Mann-Whitney U-Test with independent sampling]. (**d**) Immunohistochemical staining of Vegfa in control and LPPR5^OE^ tumors using equal exposure settings [scale bar represents 50 µm]. (**e**) Immunohistochemical staining of blood vessels (CD31) in control and LPPR5^OE^ tumors [scale bar represents 50 µm]. (**f**) Automated Analysis of blood vessel parameters show significant different diameter (**** *p* < 0.0001), vessel circumfluence, edge length (**** *p* < 0.0001), and area (**** *p* < 0.0001). No difference was found in the total number of vessels. ns = non significant.

We evaluated the potential interaction of glioma cells and endothelial cells in vivo using intrastriatal stereotactic tumor cell implantation. Consecutive weekly T1 with a contrast agent, and the T2-weighted MRI scans showed decelerated tumor growth of the LPPR5^OE^ tumors (Figure 3a). The tumor volume of the control GL261 and LPPR5^OE^ tumors differed significantly 14 and 21 days post inoculation (Figure 3b). We dissected the tumor tissue and performed qPCR screening to identify the molecular signals contributing to this drastic reduction in tumor growth. Vegfa was significantly downregulated in combination with the tumor cell–endothelial cell migration integrin Cdh2. Hypoxia (Hif1a) was unchanged. The chemoresistance-associated cytokine Cdk6 and the apoptosis-inducing membrane receptor Fas, as well as its ligand (Fasl), were significantly upregulated [13]. Additionally, the extracellular matrix remodeling molecule matrix metallopeptidase 9 (MMP9) was significantly upregulated in the LPPR5^OE^ glioma (Figure 3c). We verified the expression and secretion of Vegfa in the control and LPPR5^OE^ tumors immunohistochemically. The LPPR5^OE^ tumors displayed reduced Vegfa staining compared with the controls (Figure 3d). To further investigate the pathophysiological consequences of this reduction, we examined the morphological differences of the CD31-positive blood vessels and discovered a reduction in the vessel diameter and the area covered by blood vessels (Figure 3e,f). The number of blood vessels was similar in the LPPR5^OE^ and control tumors (Figure 3e,f).

### 2.4. Sunitinib Therapy Does Not Decelerate Tumor Growth or Further Promote Apoptosis in LPPR5OE Tumors

**Figure 4:**
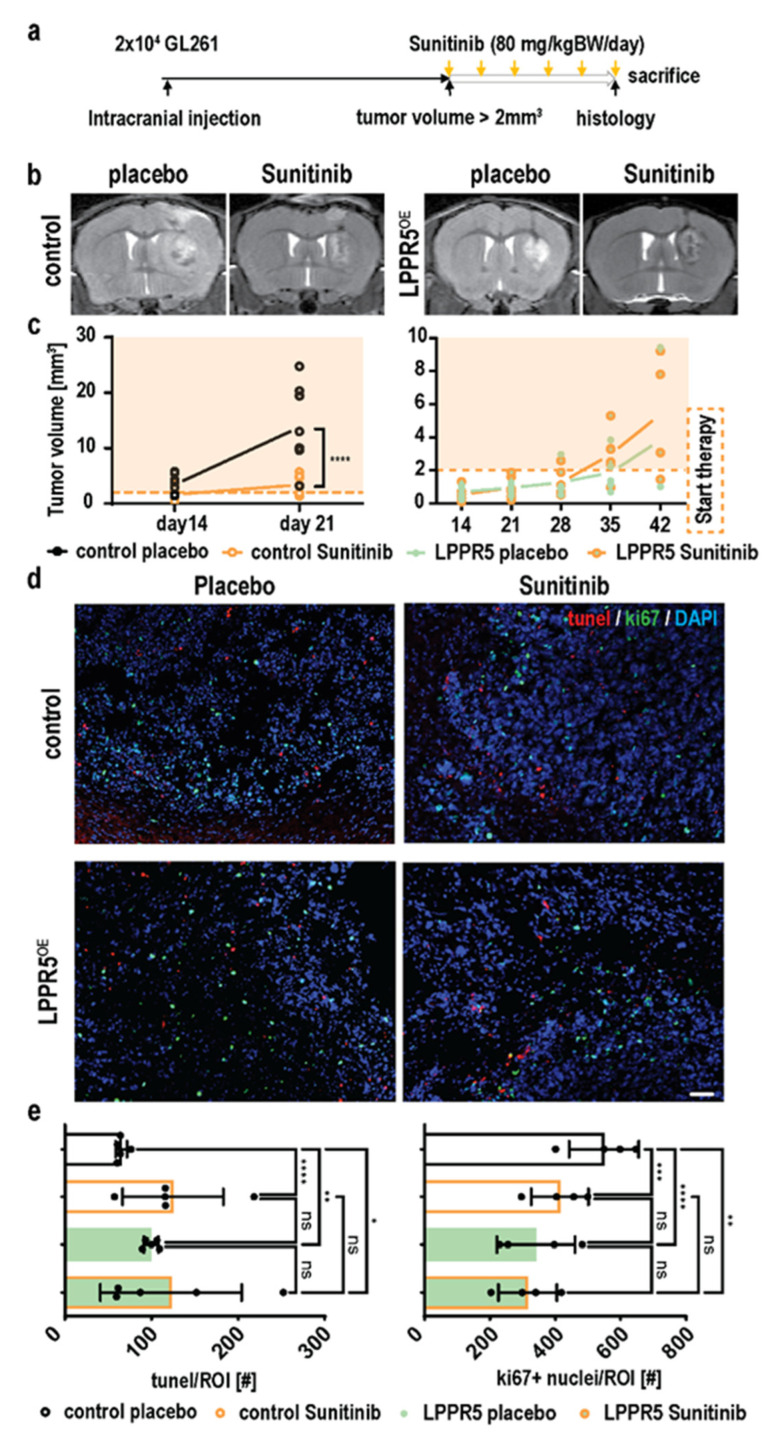
Response to antiangiogenic therapy in control GL261 and LPPR5OE tumors. (**a**) Experimental overview of the therapy experiments performed. (**b**) Visualization of the effect of sunitinib therapy on tumor growth in controls and LPPR5OE tumors. (**c**) T2 volumetric analysis of control (*p* < 0.0001) and LPPR5OE (not significant) tumors receiving placebo and sunitinib therapy. Orange shadow indicates tumors in therapy (mixed effect analysis and Sidak’s multiple comparisons test). (**d**) Immunohistochemical staining of tumor tissue for apoptosis (tunel) and proliferation (Ki67) markers. Cell nuclei are marked with DAPI staining (scale bar indicates 50 µm). (**e**) Quantification of tunel- and Ki67-positive nuclei showed significant increased apoptosis in LPPR5OE and the control therapy group (Control placebo vs. control sunitinib **** *p* < 0.0001, control placebo vs. LPPR5OE placebo ** *p* = 0.0022, and control placebo vs. LPPR5OE sunitinib * *p* = 0.0106). LPPR5OE and sunitinib therapy tumors showed a significant reduction in proliferation (Control placebo vs. control sunitinib *** *p* = 0.0005, control placebo vs. LPPR5OE placebo **** *p* < 0.0001, and control placebo vs. LPPR5OE sunitinib ** *p* = 0.0014) with two-way ANAOVA analysis and Sidak’s multiple comparisons test. ns = non significant.

We hypothesized that Vegfa downregulation in LPPR5^OE^ glioma may influence the adaptive response to antiangiogenic therapy. Antiangiogenic therapy was initiated when the threshold tumor volume of 2 mm^2^ measured by T2 MRI was reached (Figure 4a), resulting in a delayed therapy window for the LPPR5^OE^ tumors (~day 35 post injection; Figure 4a). As described previously, sunitinib treatment led to significant tumor growth reduction in the control tumors [14]. However, LPPR5^OE^ tumor growth was not reduced in the sunitinib-treated animals (Figure 4b,c). Tunel staining showed increased apoptotic activity in the LPPR5^OE^ tumors (Figure 4d,e). Sunitinib treatment of the control tumors increased intratumoral apoptosis to the level observed in the LPPR5^OE^ tumors. Antiangiogenic therapy for LPPR5^OE^ tumors, on the other hand, had no additional effect on the already elevated apoptotic activity (Figure 4d,e). Since an increase in apoptosis can be compensated by an increase in proliferation, we simultaneously stained for the proliferation marker Ki67 (Figure 4d,e). Proliferation was significantly reduced in LPPR5^OE^ compared with the control tumors. Again, sunitinib reduced cell proliferation in the controls but had no significant impact on proliferation in the LPPR5^OE^ tumors (Figure 4e).

These results motivated us to research the microvascular circulation of LPPR5^OE^ tumors in more detail to identify differences in tumor angiogenesis that could explain this unexpected resistance to antiangiogenic therapy.

### 2.5. Dysfunctional and Susceptible to Sunitinib Therapy

**Figure 5:**
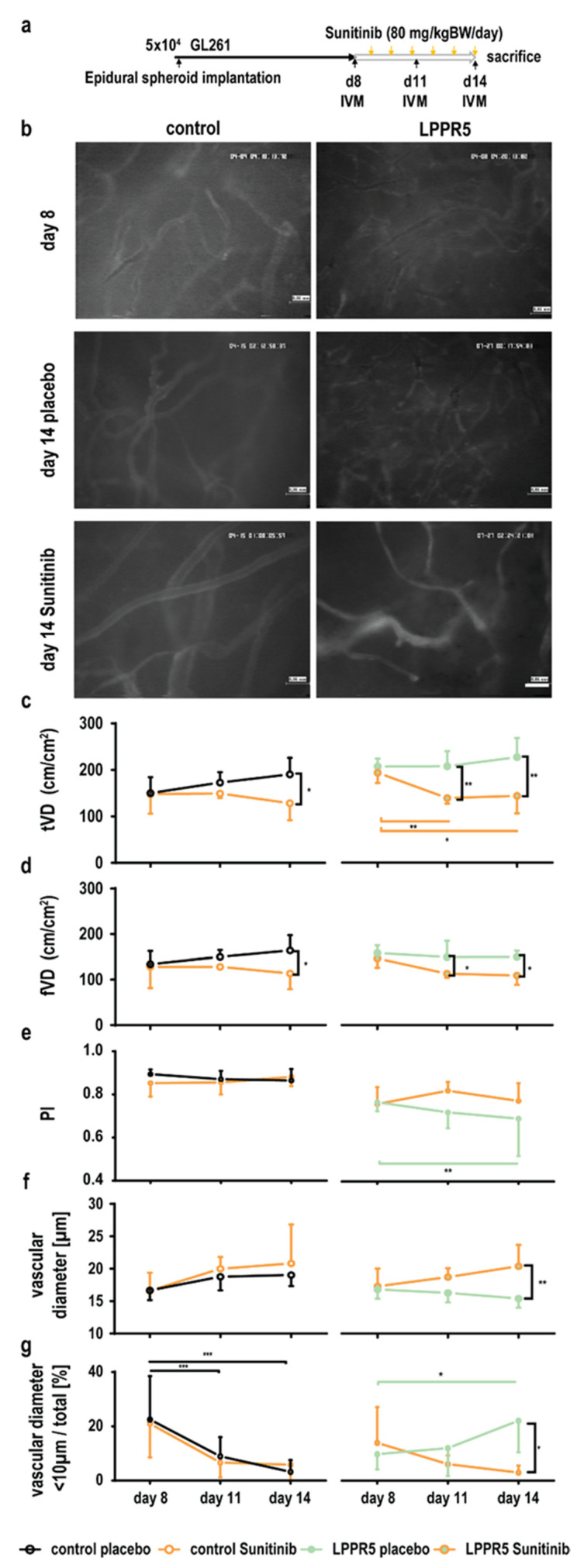
Intravital fluorescence microscopy visualization of LPPR5^OE^ vasculature. (**a**) Experimental set-up. (**b**) Exemplary images of vascular architecture in control and LPPR5^OE^ tumors 8 and 14 days after tumor cell implantation with placebo or sunitinib therapy, respectively (scale bar represents 60 µm). (**c**) The total vascular density (tVD) quantified in control and LPPR5^OE^ tumors found a significant therapeutic difference in control tumors at 14 days (* *p* = 0.0108) and a significant difference at 11 (** *p* = 0.0030, orange bar ** *p* = 0.0089) and 14 days (** *p* = 0.0014, orange bar * *p* = 0.0281) in LPPR5 tumors. (**d**) Functional vascular density (fVD) quantified in control and LPPR5 tumors showed a significant therapeutic difference in control tumors after 14 days (* *p* = 0.0300) and a significant difference after 11 (* *p* = 0.0439) and 14 (* *p* = 0.0436) days in LPPR5 tumors. (**e**) Perfusion index (PI, fVD/tVD) showed a significant difference in the LPPR5^OE^ group at 14 days (green bar ** *p* = 0.0041). (**f**) Diameter measurements found a reduction in the average diameter in the placebo-treated LPPR5 group compared with the sunitinib-treated LPPR5 at 14 days (** *p* = 0.0084). (**g**) The fraction of vessels smaller than 10 µm significantly decreased in the control tumors (11 days: *** *p* = 0.001; 14 days: *** *p* = 0.0002). LPPR5^OE^ tumors showed an increase in vessels smaller than 10 µm 14 days after tumor implantation (* *p* = 0.0194, green bar * *p* = 0.0190) (**c**–**g**): two-way ANAOVA analysis, with Sidak’s multiple comparisons test used for all statistics.

We established an intravital microscopy protocol to observe LPPR5^OE^ and control tumors with and without antiangiogenic therapy (Figure 5a). The LPPR5^OE^ tumor vessels were smaller and more susceptible to sunitinib, which resulted in a rapid reduction of the total vascular density that exceeded the levels of sunitinib-induced changes observed in the controls (Figure 5b). The total vascular density (tVD) and functional vascular density (fVD) were significantly decreased in the LPPR5 tumors 2 days after therapy (Figure 5c,d). In the control GL261 tumors, antiangiogenic therapy was significantly effective after 5 days of sunitinib therapy. Perfusion constantly decreased in the LPPR5 tumors (Figure 5e). Simultaneously, we found a constant reduction in vascular diameter in these tumors over time (Figure 5f). We analyzed this further by comparing the ratio between the number of vessels with diameters smaller than 10 µm to the total number of tumor vessels visible in the field. A stark increase in these smaller vessels was found in the LPPR5^OE^ tumors treated with sunitinib (Figure 5g).

## 3. Discussion

We identified the downregulation of the pro-neuronal, six-transmembrane protein LPPR5 with increasing malignancy in the human glioma. More precisely, the expression was reduced in the mesenchymal glioma subtypes compared with the classical and proneural subtypes. Interestingly, the neural subtype also showed significant downregulation of LPPR5. Proneural glioma are defined by the expression of the neurogenesis markers PDGFRA, NKX2-2, and OLIG2, and they are IDH1 mutants [15]. Neural glioma are classified by NEFL, GABRA1, SYT1, and SLC12A5 expression. In normal brain tissue, LPPR5 expression is closely associated with the proneural makers across individual brain regions, with neural makers clustering differently (Figure S1b). We therefore assumed that LPPR5 expression was regulated by similar transcription factors in the proneural glioma, neural tumors, and physiological tissue. If or how LPPR5 expression is altered by IDH mutations remains a subject of further study.

We established a murine LPPR5 overexpression GL261 glioma model (LPPR5^OE^) with reduced malignancy in vivo. Unfortunately, syngeneic orthotopic murine glioma models are scarce. The GL261 model used in the current study represents the most extensively characterized model to date [16]. Given increased evidence of peripheral and CNS immune cell contributions in glioma development, more murine glioma-like cell lines are needed [17,18].

We identified a pro-apoptotic, slowly proliferating LPPR5^OE^ tumor with dysfunctional microcirculation in vivo. The pro-apoptotic and vasculature phenotype associated with LPPR5^OE^ glioma provided a compelling narrative explaining the more benign behavior. Molecular screening and immunohistochemistry identified a reduction in Vegfa expression in LPPR5^OE^ tumors in combination with unchanged expression of Hif1α compared with control tumors four times their size. We assumed the LPPR5^OE^ tumors were under constant, size-independent hypoxic stress and therefore experienced a significant increase in apoptotic events. This hypoxia-induced apoptosis was mediated by an increase in Fasl release in combination with elevated Fas expression in the LPPR5^OE^ tumors [19]. The primary cause of this hypoxia was the reduced Vegfa expression and secretion, resulting in a smaller neovasculature incapable of delivering adequate nutrients. Given the increased susceptibility of small vessels to antiangiogenic therapy, we hypothesized an additional benefit regarding tumor growth reduction under antiangiogenic therapy [20].

Murine Vegfa is not sufficiently immuno-neutralized by the human Vegfa antibody bevacizumab, which is used for antiangiogenic therapy [21]. However, Vegfa receptor inhibition by sunitinib replicated a well-described antiangiogenic effect in the murine glioma [14,20]. The effects of sunitinib on the pruning and normalization of the neovasculature in the LPPR5^OE^ tumors exceeded the sunitinib effects in the controls. Unfortunately, antiangiogenic therapy had no additional effect on the existing hypoxia-induced apoptosis or proliferation of LPPR5^OE^ tumors, as the effects of sunitinib observed in the GL261 glioma were possibly a mixture of antiangiogenic and pro-apoptotic drug actions [14,22]. The pro-apoptotic phenotype of the LPPR5^OE^ tumors could explain the failure of sunitinib therapy regarding macroscopic growth reduction. Moreover, the placebo-treated LPPR5^OE^ tumors failed to grow exponentially after reaching the therapy threshold size of 2 mm^3^, and a reduction in size might not be detectable due to a slice width of 0.5 mm and a lesion size of 2–5 mm^3^. Antiangiogenic and chemotherapeutic agents have been shown to be most effective when administered during the phase of exponential growth [23,24]. Hence, we hypothesize that the increase in hypoxia with antiangiogenic therapy had no additional effect on tumor growth given the already slow hypoxia-adapted growth of the LPPR5^OE^ tumors.

These findings uncover a novel pathway for the modulation of chemo- and antiangiogenic therapeutic sensitivity. Previously published in vitro data on modulating LPPR5 signaling pathways in glioma show the involvement of ROCK kinase [25,26]. Downregulation of ROCK2 in U251 cells increased the cytotoxic sensitivity to the alkylating chemotherapeutic agent temozolomide [25]. Downregulation of ROCK1 in rat glioma cells increased the sensitivity to the anti-neoplastic agent ACNU (nimustin) [26]. Additionally, we identified an endothelial feedback signal in vitro, by which the GL261 glioma cells increased LPPR5 expression after treatment with the endothelial cell conditioned medium. The promoter region controlling LPPR5 expression has not yet been characterized. In silico analysis identified eight potential transcription factor sites (GeneHancer ID: GH01J099005). The early growth response 1 (EGR1) binding site is of particular interest, as EGR1 is a downstream target of insulin-like growth factor II (IGF-II) [27]. Two independent screens have identified IGF-II and other insulin-like growth factors in an endothelial cell conditioned medium [28,29]. Therefore, the increase of LPPR5 expression in the control GL261 cells after treatment with a conditioned medium was potentially mediated by the IGF-II–EGR1 pathway. Molecular promoter activation assays are needed to research this in more detail.

Multiple growth factors, endosomes, cytokines, and proteinases are present in endothelial conditioned media, which could lead to the increased migration observed in the GL261 glioma cells [28,29,30,31,32,33]. This migration was further potentiated in the LPPR5^OE^ clones, indicating a receptor or receptor modulation function by LPPR5. However, no pro-invasive phenotype was found in the LPPR5^OE^ tumors in vivo. Glioma invasion may be compromised by a reduction in Cdh2 expression in vivo, an important molecule for tumor cell–endothelial cell adhesion [34]. Downregulation disturbs the preferred route of glioma cell invasion along cerebral blood vessels [35]. Furthermore, the evaluation of the immune system response could provide additional insight into the migratory discrepancy found between the in vitro and in vivo paradigms (Figure S3) [36].

Although most human glioma cell lines show low LPPR5 expression, patient survival did not significantly correlate with LPPR5 expression (Figure 1a and Figure S4). It is therefore not clear if pharmacological interventions targeting LPPR5 or its upstream regulators will prolong glioma patient survival. Furthermore, the molecular mechanism underling LPPR5-regulated Vegfa release remains elusive and should be investigated more deeply.

## 4. Materials and Methods

### 4.1. In Silico Data Collection and Analysis

For the expression data on physiological LPPR5 expression, the GTEx and GEO datasets were used. The NIH Genotype-Tissue Expression (GTEx) project is a sample data resource for studies on the relationship between genetic variation and gene expression in multiple human tissues supported by the Common Fund of the Office of the Director of the National Institutes of Health, NCI, NHGRI, NHLBI, NIDA, NIMH, and NINDS (dbGaP Accession phs000424.v7.p2, 28.02.2009) [37]. The Gene Expression Omnibus (GEO) platform (EDGAR, GEO, 2002) was used to obtain RNA-Seq data generated by Duff et al. from human tissues on 13 January 2018 [38]. For data on LPPR5 in glioma, the cBioPortal was accessed on 9 February 2018 and 28 April 2018. cBio is an open-access resource, and it was used to evaluate the TCGA provisional datasets from the TCGA datacenter [39,40,41]. Consequently, the results here were in part based upon data generated by the TCGA Research Network. Available online: http://cancergenome.nih.gov (accessed on 28 February 2009). Additional data were obtained from Oncomine, a commercial platform owned by ThermoFisher used to access the Sun brain dataset for expression data and the mostly matched Kotliarov dataset for copy number alterations, as well as the Freije brain library dataset for detailed survival data, on 4 September 2018 [12,42,43,44].

### 4.2. Cell Culture

GL261, N2A, and BEnd4 cells were cultivated in DMEM, high glucose, glutaMAX, 10% FBS, and 100 U/mL penicillin-streptomycin at 37 °C and 5% CO_2_. A conditioned medium was generated as previously described [45]. Cell viability and trans-well migration were measured using the CELLigence RTCA DP system (Agilent Technologies, Ratingen, Germany) according to the manufacturer’s instructions [46]. We used the GL261 tumor cell line from C57BL/6 mice for both the orthotopic implantation and the chronic cranial window (ACC 802, DSMZ, Braunschweig, Germany). The coding sequence of rat LPPR5 was fused with red fluorescent protein (pRFP-N1 vector, kindly provided by Dr. Stefan Rothenburg, NIH, Bethesda, MD, USA) and introduced into the cells with a lipofectamine transfection (Invitrogen, Karlsruhe, Germany) reagent according to the manufacturer’s protocol. The rat LPPR5 amino acid sequence differs from the mouse sequence at two loci (N188S and I285V). However, mouse and rat sequences induce similar phenotypes in culture [9,47]. We used fluorescence-activated cell sorting (FACS) for RFP at the Flow Cytometry Core Facility (Deutsches Rheumaforschungszentrum DRFZ, Berlin, Germany) to select the top 10% expressing tumor cells. The tumor cells were cultivated in a monolayer to a maximum of 80% confluency using G418 (600 µg/mL) as a selection agent. The cell count and cell vitality were assessed using the CASY Cell Counter TT (OLS). The cells were thawed 5 days prior to implantation and passaged 2 days prior to implantation. The control cells, transfected with the empty vector construct, received identical treatment.

### 4.3. Immunoblotting

Western blotting was performed as previously described [9]. Briefly, the cells were lysed in an RIPA buffer containing protease inhibitors. The protein concentration was measured according to the manufacturer’s instructions (#23227, Thermo Fisher, Dreieich, Germany). The cell lysates were resolved on a 2–14% SDS-PAGE gel and transferred onto a nitrocellulose membrane. The membranes were blocked with 5% BSA in TBS + 0.05% Tween 20 for 1 h at room temperature. The primary antibodies were incubated overnight at 4 °C. JL-8 monoclonal mouse anti-GFP antibody (1:1000, #632380, Takara Bio, Saint-Germain-en-Laye, France) was used to detect a conserved amino acid sequence in GFP-derived fluorophores included in the RFP. An HRP-conjugated donkey anti-mouse antibody was used as a secondary antibody (1:10,000, #115-035-003, Jackson ImmunoResearch, Ely, UK). For loading control, an anti-actin-HRP antibody (1:10,000, #A3854, Sigma-Aldrich, Darmstadt, Germany) was used (incubation: 1 h at room temperature). An ECL reaction was performed using a SuperSignal Femto Substrate (#34095, Thermo Fisher, Dreieich, Germany).

### 4.4. Orthotopic Intrastriatal Implantation Model

Orthotopically implanted GL261 cells were described to form tumors with high grade glioma characteristics in immunocompetent mice [16,36,48,49]. The 8–12-week-old C57Bl6/J were obtained from our in-house animal facilities. General anesthesia was induced by an i.p. injection of 70 mg/kg Ketaminhydrochlorid (Ketavet) and 16 mg/kg Xylazine (Rompun 2%, Bayer, Berlin, Germany) dissolved in sterile water. After positioning in a stereotactic holder, a longitudinal incision was made by drilling a 23G burr hole, 1 mm rostral, and 2 mm lateral of bregma. A Hamilton syringe loaded with 2 × 10^4^ tumor cells in 1 µL PBS was lowered 4 mm into the brain parenchyma and then retracted by 1 mm. The tumor cells were incrementally injected over 5 min. After the injection, the syringe was retracted slowly over 5 min. The incision was closed with single sutures, and the mice were placed on a heating pad until regaining consciousness. The mice were perioperatively injected with Phenoxymethylpenicillin (5 Mega, InfectoCilin, InfectoPharm, Heppenheim, Germany) intramuscularly, and the drinking water was enriched with tramadol for the first two postoperative days.

### 4.5. MRI Quantification of Tumor Growth Dynamics

Weekly volumetric measurements of the tumor mass and edema were performed with Paravision 5.1 using a 7 Tesla rodent MRI (PharmaScan 70/16, Bruker BioSpin MRI GmbH, Billerica, MA, USA). The imaging protocol consisted of a T2-weighted and T1-weighted sequence, the latter with gadopentetate dimeglumine (Magnevist, Bayer, Berlin, Germany) as a contrast agent and each with 20 coronal slices per sequence and a 0.5-mm thickness per slice (T2 sequence: TR/TE54200/36ms, Rare factor 8, 4 averages; post-contrast T1: TR/TE5800/10.ms, Rare factor 2, 4 averages), showing the brain from the olfactory bulb to the cerebellum. The field of view was 2.56 × 2.56 cm with a matrix size of 256 × 256 and a nominal voxel size of 98 µm × 98 µm. The animals received isoflurane inhalation anesthesia (1.5% for maintenance and 2.0% for induction in 70% N_2_O and 30% O_2_ via a facemask) for the duration of the scan (approximately 20 min). The respiration rate was monitored continuously (Small Animal Instruments, Stony Brook, NY, USA) and maintained at 100/min to control narcosis. The body temperature was maintained using a heat pad. Volumetric analysis was performed using Analyze 10.0 (Analyze Direct, Inc., Lenexa, KS, USA) and ImageJ 1.53 (NIH, Bethesda, MA, USA) software. Contrast-enhancing areas in the T1-weighted sequences and T2-hyperintese areas were marked with a region-of-interest tool, converted into a 3D map of the tumor, and used to calculate the volume automatically. The delta between the T1 and T2 volumes was considered the peritumoral edema, potentially accompanied by diffuse infiltration [50].

### 4.6. Antiangiogenic Treatment: Sunitinib Therapy

The murine experiments included placebo-treated and Sunitinib-treated groups. High-dose weight-adapted (80 mg/kg bodyweight) Sunitinib dissolved in dimethyl sulfoxide (DMSO) was administered intraperitoneally (i.p.) for 6 or 5 consecutive days as described previously [20] (Figure 4a and Figure 5a). The placebo-treated animals were injected intraperitoneally with a weight-adapted volume of DMSO for the same duration. Although data on the optimal starting points for Sunitinib therapy in murine glioma models are scarce, previous research on temodal in murine glioma models suggests optimal efficacy for tumor growth in protocols where therapy is administered during the period of growth rather than immediately after tumor cell implantation, while an early start of therapy leads to better overall survival [23]. The administration of Sunitinib after reaching a threshold tumor size emulated clinical reality better than starting therapy immediately after implantation. Accordingly, the starting point of placebo or Sunitinib therapy for each group was the day after reaching a threshold median tumor size of 2 mm^3^. After completion of therapy, the animals were either perfused with a 4% paraformaldehyde solution and decapitated or sacrificed without perfusion. The former group was used for immunohistochemistry, the letter for RNA extraction, and qPCR.

### 4.7. Immunohistochemical Staining

For immunohistochemistry, the brains were dissected, cryoprotected in 40% sucrose solution, frozen in isopentane, and stored at −80 °C. The brains were mounted in a TissueTek (Sakura, Umkirch, Germany) block in an a.p. orientation and, 20-µm coronary sections were cut with a cryostat (Microm Cryo-Star HM 560, Thermo Fisher, Dreieich, Germany) at −16 to −24 °C, mounted on Superfrost microscope slides (R. Langenbrink, Emmendingen, Germany), and stored at −80 °C until the immunofluorescent staining was performed. After thawing, the sections were washed in PBS (3 × 5 min) and blocked in 1% Casein in PBS for 30 min at room temperature. After two additional washes with 0.5% Casein/PBS, the slices were incubated with the following primary antibodies, diluted in 0.5% Casein at +8 °C overnight: rabbit anti-Ki67 (1:200, clone SP6, Thermo Fisher, Dreieich, Germany) [51], rabbit anti-LPPR5 (1:500, SAB4301184, Sigma Aldrich), rabbit polyclonal anti-Vegfa antibody (1:100, ab46154, Abcam, Berlin, Germany), rat anti-CD31 (1:50, 550274, BD Biosciences, Heidelberg, Germany), mouse anti-SMA (1:100, A5228, Sigma-Aldrich, Darmstadt, Germany), and mouse anti-NeuN (1:200, MAB377, MilliporeSigma, Burlington, MA, USA). The following day, after 3 × 5min washing in PBS, the following fluorescently labeled secondary antibodies were applied and incubated for 2 h at RT: Cy5-conjugated (1:200, 711-175-152, Jackson ImmunoResearch, Ely, UK), Cy3-conjugated (1:200, 712-165-153, Jackson ImmunoResearch, Ely, UK), and Alexa647-conjugated (1:200, 711-605-152, Jackson ImmunoResearch, Ely, UK). After further washing, the nuclei were stained with 4′,6-diamidino-2-phenylindole (DAPI), washed, and covered using Immu-Mount (Thermoscientific, Waltham, MA, USA). The Apoptaq kit (MilliporeSigma, Burlington, MA, USA) was used to quantify the apoptosis within the tumor according to the manufacturer’s instructions [52,53]. These TUNEL-stained slides were counterstained with anti-Ki-67-antibody/Cy5 and DAPI as described above. Microscopic evaluation of the slices was performed using a Zeiss fluorescent microscope (Obeserver Z1, 5× EC Pln N, 5×/0.16 DIC0, resolution: 2.0 µm, 10× Pln Apo, 10×/0.45 DIC II, resolution: 0.74 µm, 20× Pln Apo, 20×/0.8 DIC II, resolution: 0.42 µm, using a HAL 100 and detectors for DAPI, GFP, DsRed, and Cy5). The pictures were captured with AxioVision. We applied a systematic approach to capture 5 pictures of the peritumoral, marginal, and central regions of the tumor each in 5 different slices of the tumor (unless the tumor was too small for this regimen), as well as whole-tumor composite images of the exemplary tumors. Analysis was carried out with Fiji 1.53c (ImageJ, NIH, Bethesda, MA, USA), where we evaluated the positive or double-positive cells or respective nuclei in the tumor area and normalized the results as positives per standard field of view. Image correlation of the DAPI and LPPR5 signal was performed with an automated CellProfiler 2.2 pipeline (Broad Institute, Boston, FL, USA) [54,55]. In this setting, correlation is defined as the correlation between a pair of images I and J, calculated as Pearson’s correlation coefficient. The formula was covariance (I, J)/[std(I) × std (J)]. We used DAPI as I and LPPR5 as J. The vascular morphology of the immunohistochemical sections was based on the CellProfiler 4.2.1 pipeline developed by Tian et al. [56]. The analysis pipelines were added to the supplemental material.

### 4.8. RNA Extraction and qPCR

Quantitative real-time PCR was performed as previously described [45]. Murine LPPR5 primers (forward = 5′-cagatgtgatagcaggcttcc-3′, reverse = 5′-catgtgacttccgcaaagg-3′) were used for the detection of LPPR5. After sacrifice without perfusion, the tumor tissue was explanted and trypsinated, and the lysates were FAC sorted for their respective fluorescent tag and stored at −80 °C. After thawing at room temperature, 200 µL of chloroform was added and vortexed to mix. After incubation (3 min at room temperature), the samples were centrifuged at 12,000× *g* for 15 min at 4 °C. After centrifugation, the upper phase with the RNA was removed into a clean tube, and 500 µL of isopropanol was added to precipitate the RNA. After incubation (room temperature, 10 min), the samples were centrifuged at 10,000× *g* for 10 min at 4 °C, and the supernatant was decanted. The precipitated RNA was washed by adding 1 mL of 70% EtOH, brief vortexing, and centrifuging at 7600× *g* for 5 min at 4 °C. The ethanol was decanted from the now-pelleted RNA, and the samples were left to air dry for 10 min and resuspended in 40 µL DEPC water. After reverse transcription using the QuantiTect Reverse Transcriptase Kit (Qiagen, Hilden, Germany) as directed by the manufacturer, the samples were immediately placed on ice and stored at −80 °C until further use. For quantitative real-time PCR of the putative targets involved in apoptosis, chemoresistance, and angiogenesis in glioma, a Qiagen QuantiFAST SYBR Green Kit and a Light Cycler 2.0 (Roche, Basel, Switzerland) were used according to the manufacturer’s instructions (initial activation at 95 °C for 5 min, denaturation at 95 °C for 10 s, and combined annealing and extension at 60 °C for 30 s for 35–40 cycles). The baseline and threshold values were set manually and kept constant across different runs for samples from the same experiment. Data analysis was performed with the 2(ΔΔCt) method with a pooled standard for target gene reference and housekeeping gene expression. Technical duplicates were performed for each experiment. The average values of the technical duplicates were plotted as individual dots.

### 4.9. Analyzing Vascular Patterns and Changes in Hemodynamics and Angiogenesis Using Intravital Epi-Illuminating Fluorescence Video Microscopy in a Chronic Cranial Window Model

The tumor cells were prelabeled with 1,1′-dioctadecyl-3,3,3′,3′-tetramethylindocarbocyanine perchlorate (DiI, 7.5 µg/mL growth medium) following the manufacturer’s instructions (Invitrogen). The cells were suspended in a culture medium containing 20 % Methocell medium (Sigma-Aldrich, Darmstadt, Germany) at a density of 500,000 cells per ml. Then, 100 µL of cell suspension per well was distributed to an uncoated, non-adhesive, round 96-well plate (Sarstedt). After 48 h, round spheroids roughly 500 µm in diameter were selected for implantation. This protocol guaranteed an equally sized tumor-like mass for the LPPR5^OE^ and control tumors for the intra-vital microscopy investigation.

For the establishment of the chronic cranial window, general anesthesia in the Bl6/J mice was induced as described above. After positioning in a stereotactic holder, longitudinal incision, and drilling of a circular hole approximately 5 mm in diameter in the central region of the calvaria anterior of the bregma, the dura mater was removed, and the spheroid was placed subdurally onto the brain surface in a macroscopically vessel-free area. The calvaria was closed by gluing on a glass plane using dental cement, and the wound was closed with single sutures. The mice were placed on a heating pad until regaining consciousness. Perioperatively, the mice were intramuscularly injected with Phenoxymethylpenicillin (5 Mega, InfectoCilin, InfectoPharm, Heppenheim, Germany), and their drinking water was enriched with tramadol for 2 postoperative days. The intra-vital evaluation of the tumor vasculature was performed on days 8, 11 and 14 after spheroid implantation. Day 8 was also the first day of sunitinib or placebo treatment.

General anesthesia for the microscopy was induced as described above, and additionally, 2% Fluoresceinisothiocyanat (FITC)-conjugated dextran (Sigma-Aldrich, Darmstadt, Germany) in 1 mL of 0.9% NaCl was administered i.v. as an intravascular contrasting agent. For the duration of the measurements, the mice were placed in a stereotactic holder. After the recordings, the sutures were closed, and the animals were placed on a heating pad until regaining consciousness. As described previously, we used the Axiotech Vario microscope (Attoarc; Zeiss, Jena, Germany) with a blue (450–490 nm) and green (520–570 nm) filter block modified with a charge-coupled device (CCD) video camera with an optional image intensifier designed to detect weak fluorescence signals (Kappa, Gleichen, Germany) [14,20,57]. The images were recorded using an S-VHS video system (Panasonic, Kadoma, Japan) for offline analysis with a computer-assisted analysis system (CapImage 8.0, CAPIMAGE-Cyntel-Software-Engineering, Heidelberg, Germany). We evaluated both the tumors’ marginal and central areas separately in 4–5 observations, acquiring 8–10 video sequences of each animal at all time points. As established by Vajkoczy et al., the microcirculation in the newly formed tumor vessels was quantified [57]. With computer assistance, the total intratumoral vascular density (TVD, cm/cm^2^, length of both perfused and non-perfused microvessels per surface area), functional intratumoral vascular density (FVD, cm/cm^2^, total length of all perfused microvessels per surface area), and blood flow in the single microvessels (Q, nL/sec) were measured. The ratio of FVD/TVD was defined as the perfusion index (PI).

### 4.10. Randomization and Statistical Evaluation

There was no randomization in the placebo or sunitinib subgroups. The animals within those groups were randomly allocated to LPPR5 overexpression or control. The group size n was ≥6 unless specified otherwise. The experimenter was blinded for the MRI-volumetry and IHC analysis, which was not feasible for intravital fluorescence video microscopy. Statistical analysis was performed with GraphPad Prism 7 (San Diego, CA, USA). For statistical testing, one-way ANOVA and two-way ANOVA with Sidak’s multiple comparison test were used. For longitudinal comparison within groups, single values of datasets with entries for each time point were used. For group comparisons, the means of all data points available were used, and *p* < 0.05 was considered significant, with significance levels indicated in the graphs by asterisks as follows: *p* ≤ 0.05 *, *p* ≤ 0.01 **, *p* ≤ 0.001 ***, and *p* ≤ 0.0001 ****. The results are presented as the mean and standard deviation unless stated otherwise.

## 5. Conclusions

The in-silico data identified an anti-oncogenic role of the membrane molecule LPPR5 in glioma. The LPPR5^OE^ GL261 tumors were characterized by a more benign phenotype with delayed growth, reduced Vegfa secretion, and a dysfunctional, predominantly small in diameter vascular architecture which showed increased susceptibility to sunitinib therapy. The lack of macroscopic growth arrest during sunitinib therapy was not mediated by vascular resistance mechanisms but likely a consequence of LPPR5^OE^ glioma hypoxia-adapted growth. This is also in accordance with the upregulation of Fas-l induced by hypoxia and predictive of a longer progression-free survival in low grade glioma [19]. Finally, we present preliminary evidence establishing LPPR5 as a predictive and prognostic biomarker in diffuse glioma for use in clinical diagnostics to identify patients that benefit from antiangiogenic therapy.

## Data Availability

The data that support the findings of this study are available from the corresponding author upon reasonable request.

## Supplementary Materials

The following supporting information can be downloaded at: https://www.mdpi.com/article/10.3390/ijms23063108/s1.
