# Supplementary material for: LPPR5 Expression in Glioma Affects Growth, Vascular Architecture, and Sunitinib Resistance
Source: Int J Mol Sci. 2022 Mar 13;23(6):3108. doi: 10.3390/ijms23063108 (PMC8952597; doi:10.3390/ijms23063108)

Supplemental Figure 1

a

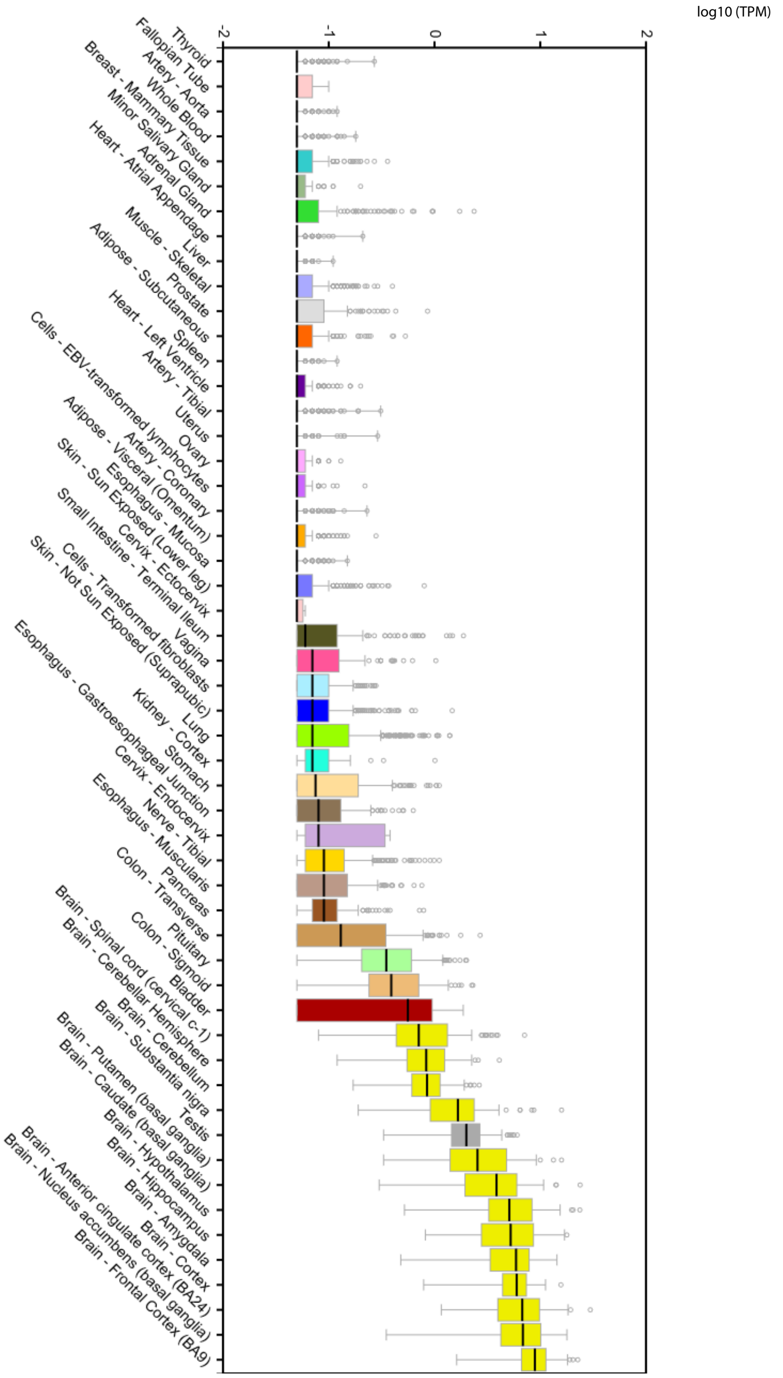

Figure S1 a) expression of LPPR5 in different organs b) LPPR5 expression in the brain compared to neural (green) and proneural (red) classification markers.

b

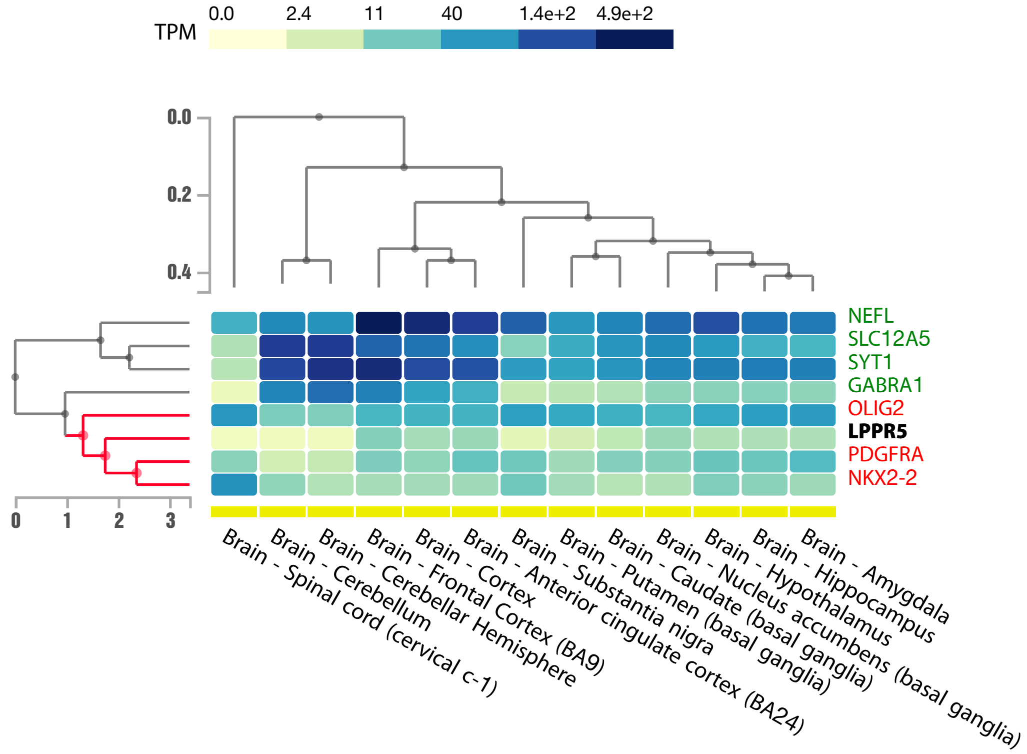

Supplement: Supplementary file 1 [file ijms-23-03108-s001.zip › SupplementalFigure 1.pdf]
