# Supplementary material for: LPPR5 Expression in Glioma Affects Growth, Vascular Architecture, and Sunitinib Resistance
Source: Int J Mol Sci. 2022 Mar 13;23(6):3108. doi: 10.3390/ijms23063108 (PMC8952597; doi:10.3390/ijms23063108)
Supplement: Supplementary file 1 [file ijms-23-03108-s001.zip › SupplementalFigure 2.pdf]

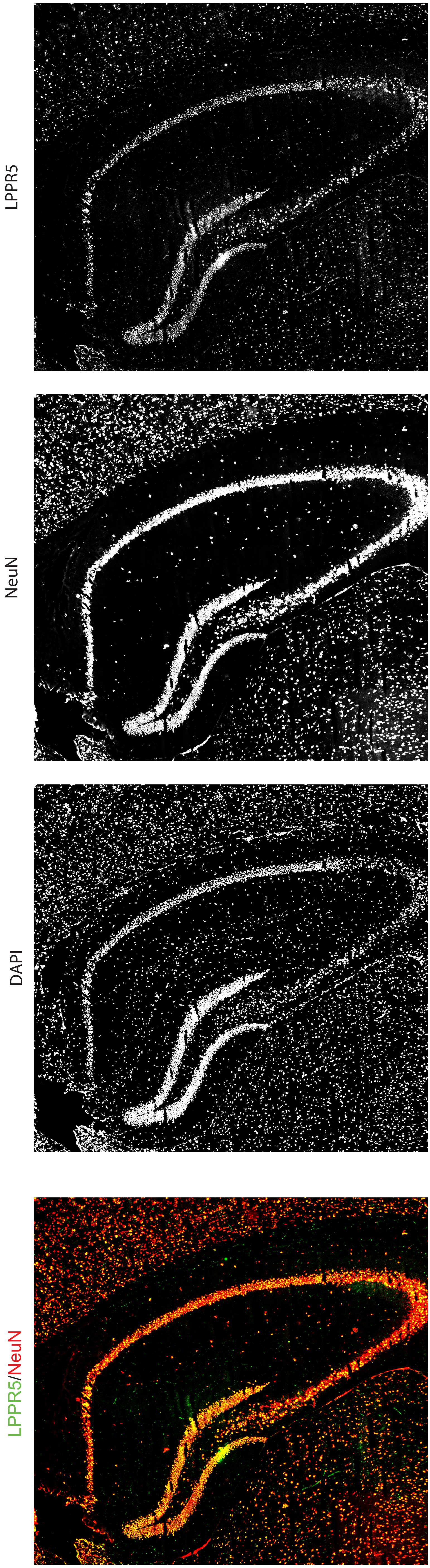

Figure S2: High resolution images of LPPR5 Immuno-
histochemical staining in the hippocampus
