# Supplementary material for: LPPR5 Expression in Glioma Affects Growth, Vascular Architecture, and Sunitinib Resistance
Source: Int J Mol Sci. 2022 Mar 13;23(6):3108. doi: 10.3390/ijms23063108 (PMC8952597; doi:10.3390/ijms23063108)
Supplement: Supplementary file 1 [file ijms-23-03108-s001.zip › SupplementalFigure 4.pdf]

Survival proportions in GBM

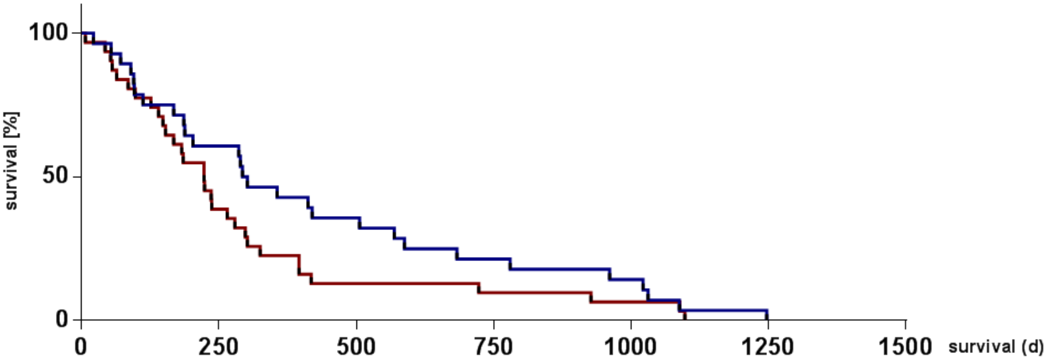

% alive				
high LPPR5	60.7	35.7	21.4	14.3
low LPPR5	38.7	12.9	9.7	6.4

p>0,05

low LPPR5 mean survival 223,0

high LPPR5 mean survival 297,5

Figure S4: Low LPPR5 mRNA expression is insignificantly associated with lower survival in GBM-patients. Kaplan-Meier survival chart using Freije Brain data accessed at Oncomine, separation expression.
